# A Single‐Cell Multiomics Pipeline Maps YBX1 as a Functional Biomarker for Immune Evasion and Therapeutic Resistance in Prostate Adenocarcinoma

**DOI:** 10.1155/humu/2147624

**Published:** 2026-05-04

**Authors:** Changcheng Luo, Dongxu Lin, Jingmin Yang, Kai Cui, Zhong Chen

**Affiliations:** ^1^ Department of Urology, Tongji Hospital, Tongji Medical College of Huazhong University of Science and Technology, Wuhan, Hubei, China, hust.edu.cn; ^2^ Institute of Urology, Tongji Hospital, Tongji Medical College of Huazhong University of Science and Technology, Wuhan, Hubei, China, hust.edu.cn; ^3^ College of Life Science and Technology, Huazhong University of Science and Technology, Wuhan, Hubei, China, hust.edu.cn

**Keywords:** immune evasion, multiomics biomarker, prostate adenocarcinoma, single-cell RNA sequencing, YBX1

## Abstract

Translating high‐resolution multiomics data into clinically actionable biomarkers is critical for overcoming therapeutic resistance and tumor heterogeneity in prostate adenocarcinoma (PRAD). To decode the complex immunosuppressive tumor microenvironment (TME) and identify robust prognostic targets, we developed a systematic biomarker discovery pipeline integrating single‐cell RNA sequencing (scRNA‐seq) mapping and high‐dimensional network analysis. By deconvoluting scRNA‐seq profiles from over 35,000 PRAD cells, nonnegative matrix factorization (NMF) of the malignant epithelial compartment revealed nine distinct transcriptional metaprograms (MPs). High‐dimensional weighted gene coexpression network analysis (hdWGCNA) pinpointed PRAD‐MP7 as the core proliferative engine and nominated the malignant‐specific gene YBX1 as the master prognostic hub. To establish clinical utility evidence, we validated YBX1 across six independent global PRAD cohorts, where its overexpression robustly predicted poor overall survival (OS) and relapse‐free survival (RFS). In vitro functional validation via siRNA‐mediated knockdown in DU‐145 and PC‐3 cells significantly attenuated proliferative and invasive capacities, impairing cell viability and downregulating key progression markers (Ki‐67, MMP2, and MMP9). Crucially, immunogenomic profiling mapped YBX1 expression to an “immune‐excluded” TME, characterized by depleted CD8+ T cell and dendritic cell infiltration alongside elevated immune checkpoint networks. Serving as a bridge to clinical translation, YBX1 effectively predicted clinical responses in three immunotherapy cohorts and demonstrated broad resistance to 12 chemotherapeutic and targeted agents. Our multiomics integration pipeline highlights YBX1 as a dual‐functional oncogene that couples malignant proliferation with immune evasion, establishing it as a highly translational biomarker and an actionable target for precision PRAD management.

## 1. Introduction

Prostate adenocarcinoma (PRAD) is the second most frequently diagnosed malignancy and the fifth leading cause of cancer‐related deaths among men globally [1]. For localized PRAD, definitive treatments such as radical prostatectomy and radiotherapy achieve favorable clinical outcomes, with a 5‐year survival rate exceeding 99%. However, once the disease progresses to advanced or metastatic stages, particularly metastatic castration‐resistant PRAD, the clinical prognosis deteriorates dramatically, with a 5‐year survival rate of less than 30% [2]. Although androgen deprivation therapy (ADT), chemotherapy, and immune checkpoint inhibitors have been applied in the clinical management of advanced PRAD, their therapeutic efficacy is largely limited by primary or acquired resistance, which is closely driven by intratumoral heterogeneity and the remodeling of the immunosuppressive tumor microenvironment (TME) [3]. Therefore, there is an urgent need to dissect the molecular mechanisms underlying PRAD malignant progression and therapeutic resistance and to identify robust prognostic biomarkers and actionable therapeutic targets.

The advent of single‐cell RNA sequencing (scRNA‐seq) technology has revolutionized our understanding of tumor biology by enabling unbiased deconvolution of the cellular architecture of the TME at unprecedented resolution [4, 5]. In PRAD, scRNA‐seq studies have revealed the complexity of TME cellular components, including diverse immune cell subsets, stromal cells, endothelial cells, and heterogeneous malignant epithelial cell populations [6]. Beyond cellular composition, the transcriptional heterogeneity within malignant cells is a core driver of tumor progression, metastasis, and treatment resistance. Nonnegative matrix factorization (NMF) has emerged as a powerful computational approach to decompose the transcriptome of malignant cells into conserved transcriptional metaprograms (MPs), which capture core biological processes underlying intratumoral heterogeneity across cancer types [7]. Meanwhile, high‐dimensional weighted gene coexpression network analysis (hdWGCNA) allows the construction of gene regulatory networks at the single‐cell level, facilitating the identification of key hub genes that govern clinically relevant malignant cell states [8]. However, few studies have integrated these approaches to systematically link proliferative transcriptional programs in malignant cells to TME remodeling, clinical prognosis, and therapeutic response in PRAD.

Y‐box binding protein 1 (YBX1), a highly conserved cold shock domain‐containing protein, participates in multiple fundamental biological processes, including transcriptional regulation, RNA splicing, DNA damage repair, and stress response [9]. Accumulating evidence has demonstrated that YBX1 acts as an oncogene in various human malignancies, promoting tumor cell proliferation, invasion, metastasis, and drug resistance [10]. Nevertheless, the functional role of YBX1 in PRAD, especially its involvement in regulating malignant transcriptional programs, TME immune landscape, and therapeutic response, has not been fully elucidated, particularly from the perspective of single‐cell intratumoral heterogeneity.

To decode the intricate interplay between intratumoral heterogeneity and immune evasion in PRAD, we executed an integrative analysis of single‐cell and multicohort bulk transcriptomes. By deconvoluting the malignant epithelial compartment, we isolated PRAD‐MP7 as the core transcriptional engine driving cancer proliferation (CP). Subsequent high‐dimensional network construction (hdWGCNA) and stringent prognostic screening nominated the malignant‐specific gene YBX1 as the master regulator of this program. Beyond validating its profound pro‐proliferative and invasive functions in vitro, our immunogenomic profiling uncovered that YBX1 actively shapes an “immune‐excluded” TME, dampening cytotoxic infiltration while upregulating immune checkpoints. Ultimately, we demonstrate that YBX1 stratification robustly predicts both clinical survival (overall survival [OS] and relapse‐free survival [RFS]) and therapeutic responses across multiple independent cohorts. Collectively, these findings establish the YBX1/PRAD‐MP7 axis as a dual‐functional driver of PRAD progression, offering a compelling prognostic biomarker and a critical vulnerability for overcoming chemo‐ and immunotherapy resistance.

## 2. Materials and Methods

### 2.1. Data Acquisition and Preprocessing

Gene expression data and corresponding clinical annotations for PRAD patients were sourced from five publicly available cohorts, comprising the TCGA‐PRAD cohort, among others [11], DKFZ2018 (https://www.cbioportal.org/), GSE107299 [12], GSE21034 [13], GSE70768 [14], and GSE70769 [14]. We obtained scRNA‐seq data from PRAD samples of GSE172316 (23,686 cells) [15] and Tabula Sapiens (12,242 cells). The R package Seurat was used to process the scRNA‐seq data [16]. The CP signature gene list was obtained from the HALLMARK database.

### 2.2. Computational Analysis

The R package InferCNV was applied to identify malignant cells within the TME. Copy number variations (CNVs) were inferred using a reference set comprising stromal and endothelial cells, and cells displaying large‐scale chromosomal aberrations were classified as malignant. Dimensionality reduction and visualization were performed using UMAP. To explore transcriptional heterogeneity within the malignant population, NMF was conducted on the cancer cell expression matrix using the GeneNMF package [7]. The optimal number of MPs was determined by evaluating the cophenetic correlation coefficient and residual sum of squares, leading to the selection of nine robust transcriptional MPs. Coexpression modules associated with PRAD‐MP7 were subsequently identified using the hdWGCNA package [8]. Functional annotation of YBX1 was conducted via the Metascape platform and Gene Set Enrichment Analysis (GSEA) of GO terms [17]. The association between YBX1 and immune cell infiltration was predicted through the MCPcounter [18], ssGSEA as described by Charoentong et al. [19], and TIMER [20]. Immunotherapy response prediction of YBX1 was performed [21]. Drug sensitivity to YBX1 was predicted using oncoPredict [22]. The Wilcoxon rank‐sum test was utilized for all drug sensitivity comparisons.

### 2.3. In Vitro Validation

Human PRAD cell lines (DU‐145 and PC‐3) were cultured in DMEM supplemented with 10% fetal bovine serum (FBS) and 1% penicillin‐streptomycin in a humidified incubator at 37°C with 5% CO_2_. Transient transfection was performed using Lipomaster 3000 Transfection Reagent to introduce either YBX1‐specific small interfering RNA (si‐YBX1) or negative control siRNA (si‐NC) into the cells. To evaluate cell proliferation, cell viability was assessed at 24, 48, and 72 h posttransfection utilizing a Cell Counting Kit‐8 (CCK‐8) assay. Additionally, DNA synthesis was quantified via an EdU incorporation assay. Cells were pulsed with EdU for 2 h, counterstained, and subsequently visualized using fluorescence microscopy. For gene expression analysis, total RNA was extracted from the transfected cells and reverse‐transcribed into cDNA. Quantitative real‐time PCR (RT‐qPCR) was then executed to determine the relative mRNA levels of YBX1, Ki‐67, MMP2, and MMP9, with target expression levels normalized to an endogenous control.

### 2.4. Statistical Analysis

Kaplan–Meier curves (log‐rank test) were used to evaluate OS/RFS. Univariate Cox proportional‐hazards regression assessed prognostic independence (HR, 95% CI). The Wilcoxon rank‐sum test compared continuous variables; Pearson’s correlation quantified linear associations. A two‐sided *p* < 0.05 was significant.

## 3. Results

### 3.1. Single‐Cell Deconvolution Delineates the PRAD TME

**Figure 1:**
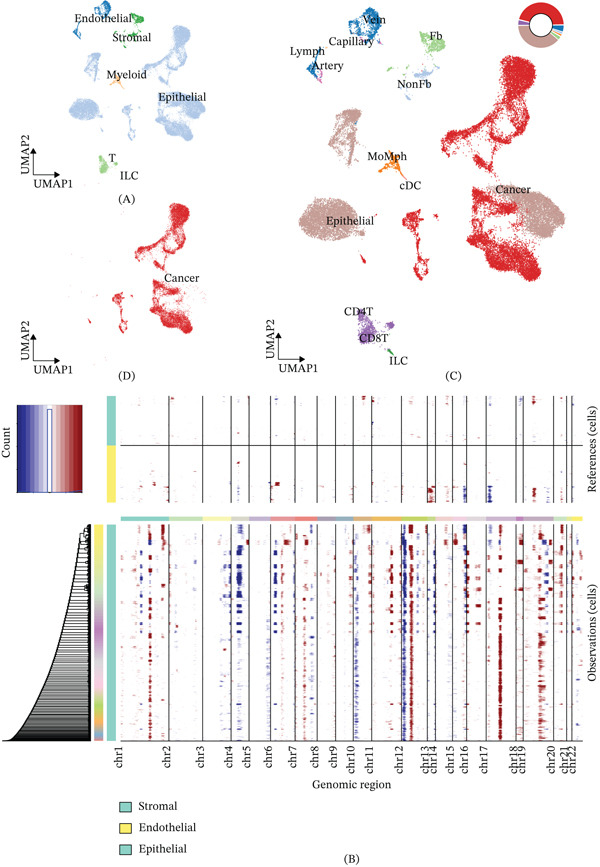
Single‐cell transcriptomic profiling and annotation of the PRAD TME. (A) UMAP visualization displaying the six major cell populations deconvoluted within the TME. (B) Heatmap illustrating inferred copy number variation (CNV) scores used to distinguish malignant epithelial cells from nonmalignant subsets. (C) UMAP plot presenting the refined minor cell types after unsupervised subclustering. (D) UMAP visualization highlighting the cancer cells within the broader TME.

We integrated scRNA‐seq profiles from multiple public cohorts to map the cellular architecture of the PRAD TME. This analytical workflow deconvoluted the TME into six major cellular compartments: T cells, epithelial cells, stromal cells, endothelial cells, innate lymphoid cells (ILCs), and myeloid cells (Figure 1A). Next, CNVs were evaluated via InferCNV (Figure 1B) to separate malignant epithelial cells from nonmalignant populations. We further refined minor cell subsets through unsupervised subclustering (Figure 1C) and visualized the malignant cells within the broader TME (Figure 1D). Finally, the identities of these refined subclusters were explicitly validated using canonical lineage‐specific marker expression profiles (Figure S1).

### 3.2. NMF Uncovers PRAD‐MP7 as the Core CP Program

**Figure 2:**
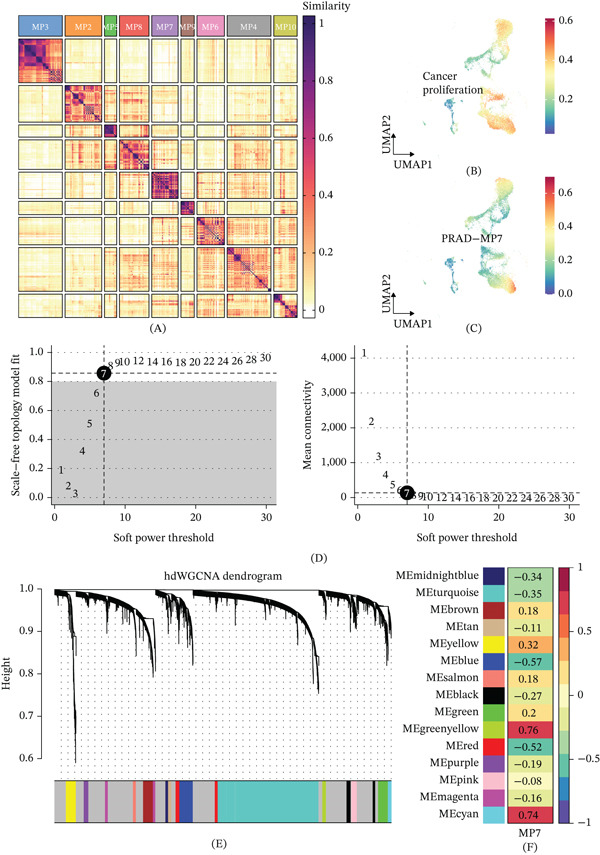
Identification of intratumoral transcriptional metaprograms (MPs) and PRAD‐MP7 regulatory networks. (A) Heatmap showing Jaccard similarity coefficients for pairwise comparisons among nine robust NMF programs in cancer cells. (B) UMAP plot illustrating cancer proliferation (CP) scores across the malignant cell population. (C) UMAP visualization of the PRAD‐MP7 signature scores in cancer cells. (D) Relationship between the scale‐free topology model fit, mean connectivity, and soft power threshold in hdWGCNA. (E) Waterfall plot demonstrating the distribution of hdWGCNA‐derived gene modules. (F) Correlation analysis matrix between the PRAD‐MP7 signature and the identified coexpression gene modules.

Focusing on the malignant compartment, we applied NMF to the cancer cell expression matrix. Based on the evaluation of the cophenetic correlation coefficient and residual sum of squares, this yielded nine robust MPs capturing intratumoral heterogeneity (Figure 2A). Among them, MP7 showed the strongest positive correlation with a well‐established CP gene signature (Figure 2B,C), pointing to PRAD‐MP7 as the primary transcriptional engine driving malignant proliferation.

### 3.3. hdWGCNA Nominates YBX1 as a Central Coexpression Node of PRAD‐MP7

To identify the upstream regulators of MP7, we performed hdWGCNA. We set a soft power threshold of 7 to maintain a scale‐free topology (Figure 2D) and partition the transcriptome into distinct modules (Figure 2E). The module eigengene (ME) of the green module correlated most strongly with the PRAD‐MP7 trait (Pearson’s *r* = 0.76; Figure 2F).

### 3.4. High YBX1 Expression Robustly Predicts Adverse Clinical Outcomes

**Figure 3:**
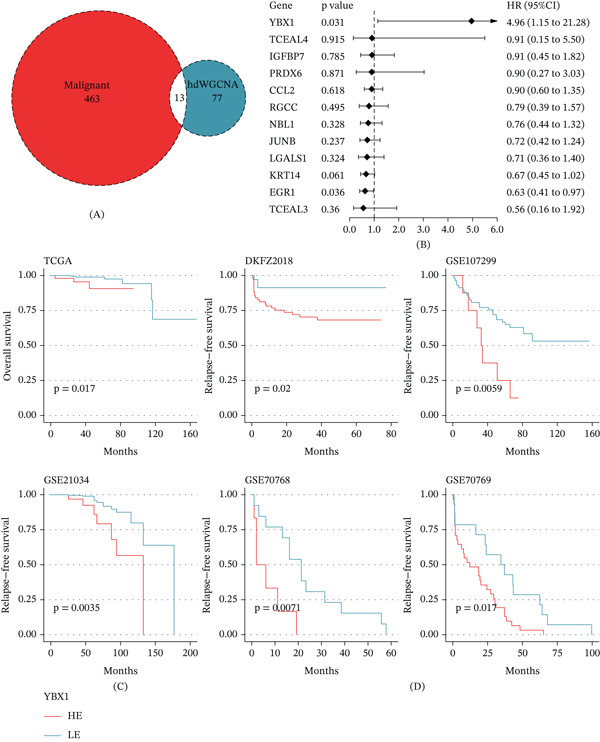
Identification of YBX1 as a master adverse prognostic gene. (A) Venn diagram illustrating the intersection between genes derived from the MP7‐associated green module and genes significantly upregulated in cancer cells. (B) Forest plot of univariate Cox regression analysis performed on the 13 intersecting candidate genes. (C) Kaplan–Meier survival curves for overall survival (OS) stratified by YBX1 expression levels in the TCGA‐PRAD discovery dataset. (D) Kaplan–Meier survival curves for relapse‐free survival (RFS) based on YBX1 expression subgroups across five independent validation datasets (DKFZ2018, GSE107299, GSE21034, GSE70768, and GSE70769).

By intersecting the 90 genes in this module with 476 transcripts significantly upregulated in malignant epithelial cells, we narrowed the candidate list down to 13 core genes (Figure 3A). Univariate Cox regression analysis then pinpointed YBX1 as the top prognostic hazard gene (Figure 3B). We subsequently evaluated YBX1’s prognostic value across six independent PRAD cohorts. In the TCGA‐PRAD discovery cohort, elevated YBX1 expression was significantly linked to shortened OS (Figure 3C). This trend held consistent across five validation cohorts (DKFZ2018, GSE107299, GSE21034, GSE70768, and GSE70769), where high YBX1 levels reliably predicted worse RFS (Figure 3D).

### 3.5. YBX1 Critically Drives Proliferative and Invasive Phenotypes In Vitro

**Figure 4:**
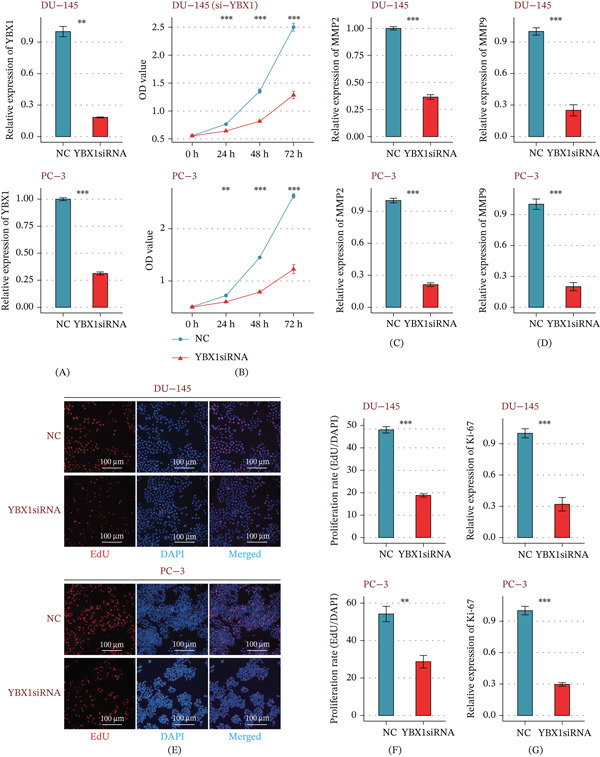
In vitro validation of the pro‐proliferative and invasive functions of YBX1. (A) RT‐qPCR assay confirming the knockdown efficiency of YBX1 in DU‐145 and PC‐3 cells following transfection with specific small interfering RNA (si‐YBX1) relative to negative control siRNA (si‐NC). (B) CCK‐8 assay results showing significantly reduced cell viability following YBX1 silencing. (C, D) RT‐qPCR analysis revealing downregulated mRNA expression of MMP2 (C) and MMP9 (D) after YBX1 knockdown. (E) Representative fluorescence micrographs of EdU staining to identify actively proliferating cells. (F) Quantification of EdU‐positive cells, indicating impaired cell proliferation after YBX1 silencing. (G) RT‐qPCR detection of reduced proliferation marker Ki‐67 expression following YBX1 knockdown. *p < 0.05, **p< 0.01,***p< 0.001; Wilcoxon rank‐sum test.

We performed siRNA‐mediated silencing in DU‐145 and PC‐3 cells to verify the functional role of YBX1. RT‐qPCR confirmed over 70% knockdown efficiency (Figure 4A). YBX1 depletion significantly impaired cell proliferation, as shown by reduced viability in CCK‐8 assays (Figure 4B) and lower expression of matrix metalloproteinases MMP2 and MMP9 (Figure 4C,D). We also observed a marked drop in EdU incorporation (Figure 4E,F) and a drastic downregulation of the proliferation marker Ki‐67 (Figure 4G). These combined results indicate that YBX1 is essential for both PRAD cell proliferation and invasion.

### 3.6. YBX1 Orchestrates an Immunosuppressive and Excluded TME

**Figure 5:**
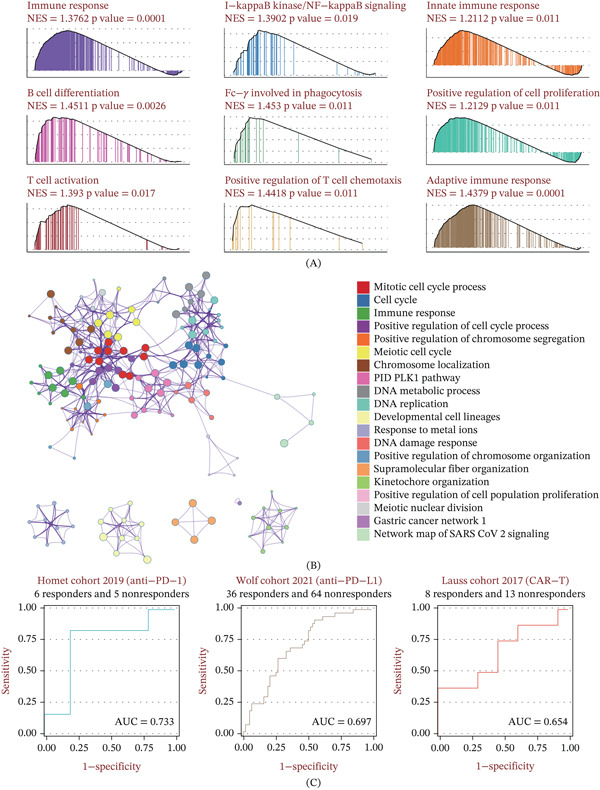
Immunogenomic enrichment characteristics of YBX1. (A) Gene Set Enrichment Analysis (GSEA) of Gene Ontology (GO) pathways associated with YBX1 expression. (B) Metascape‐based functional network enrichment analysis of pathways linked to YBX1. (C) Receiver operating characteristic (ROC) curves evaluating the predictive performance of YBX1 in three independent immunotherapy cohorts.

**Figure 6:**
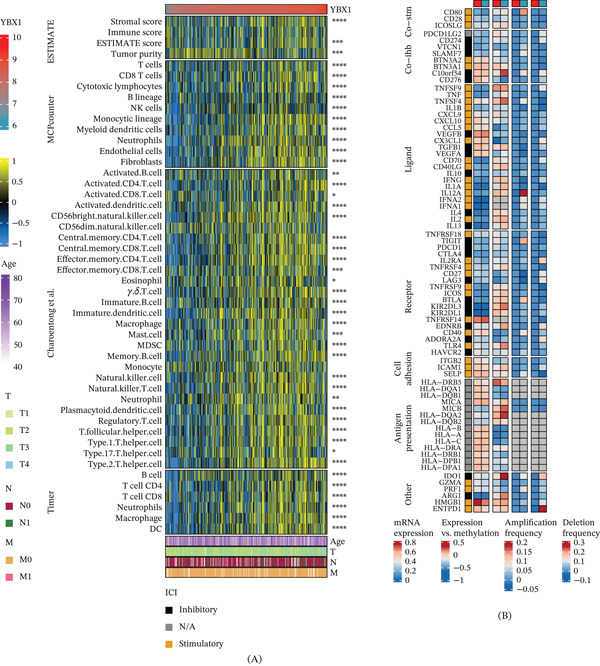
High YBX1 expression defines an immune‐excluded TME. (A) Heatmap showing the correlation between YBX1 expression and ESTIMATE microenvironment scores, as well as immune cell infiltration levels quantified by MCPcounter, TIMER, and ssGSEA using Charoentong et al.’s signatures. (B) Heatmap illustrating the positive correlation between YBX1 expression and key immune modulators. *p < 0.05, **p < 0.01, ***p < 0.001, ****p < 0.001; Pearson′s correlation test.

GSEA demonstrated that high YBX1 expression exhibited a negative normalized enrichment score (NES) for immune‐activating pathways, such as T cell activation and receptor signaling (Figure 5A). Metascape profiling corroborated the suppression of both innate and adaptive immune processes (Figure 5B). Importantly, YBX1 levels effectively predicted therapeutic responses across three immunotherapy cohorts (AUCs: 0.733, 0.697, and 0.654) (Figure 5C). At the TME level, ESTIMATE algorithm scores revealed that YBX1 was inversely associated with stromal and immune scores but positively correlated with tumor purity, a hallmark of an immunologically “cold” tumor. Deconvolution analyses (MCPcounter, ssGSEA, and TIMER) consistently linked YBX1 overexpression to reduced infiltration of CD8+ cytotoxic T cells, dendritic cells, and neutrophils (Figure 6A). Concurrently, YBX1 expression positively correlated with key immune checkpoints, including PD‐L1 and CTLA‐4 (Figure 6B).

### 3.7. YBX1 Indicates Broad Chemotherapeutic and Targeted Agent Resistance

**Figure 7:**
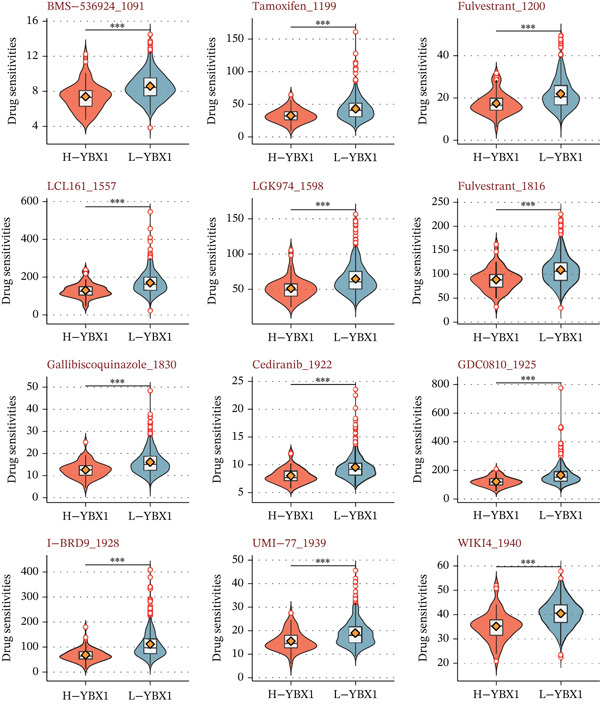
Prediction of drug response associated with YBX1. Violin plots illustrating the estimated drug sensitivity levels (IC50) of 12 chemotherapeutic and targeted agents in subgroups stratified by high (H‐YBX1) and low (L‐YBX1) expression ( ^∗^ *p* < 0.05,  ^∗∗^ *p* < 0.01,  ^∗∗∗^ *p* < 0.001; Wilcoxon rank‐sum test).

Drug sensitivity profiling revealed that high YBX1 expression correlates with significant resistance to 12 pharmacological compounds, encompassing various cell‐cycle and targeted kinase inhibitors (Figure 7). This suggests that YBX1‐driven tumors possess a multidrug‐resistant phenotype, underscoring the clinical necessity for YBX1‐targeted strategies in advanced PRAD.

## 4. Discussion

In this study, we integrated single‐cell and bulk transcriptomic data from multiple independent PRAD cohorts to systematically dissect the TME cellular landscape and malignant cell transcriptional heterogeneity. We identified a proliferation‐related transcriptional MP7 and nominated YBX1 as a key regulatory gene of this program. Through multicohort prognostic validation, in vitro functional experiments, and comprehensive immunogenomic analysis, we demonstrated that YBX1 acts as an oncogenic driver to promote PRAD cell proliferation and invasion, shape an immunosuppressive TME, and predict poor response to immunotherapy and chemotherapy. Our findings not only reveal the critical role of YBX1 in PRAD malignant progression but also provide a potential biomarker and therapeutic target for the clinical management of PRAD.

First, our study delineated the comprehensive cellular architecture of the PRAD TME at single‐cell resolution. Through integrated analysis of scRNA‐seq datasets, we resolved six major cellular compartments, including T cells, epithelial cells, stromal cells, endothelial cells, ILCs, and myeloid cells, which is consistent with previous single‐cell studies of PRAD [15]. Using InferCNV analysis, we accurately distinguished malignant epithelial cells from nonmalignant cell populations, which laid a reliable foundation for the subsequent dissection of intratumoral transcriptional heterogeneity. NMF decomposition of malignant cell transcriptomes identified nine robust transcriptional MPs, among which MP7 showed the strongest correlation with the CP signature. This finding indicates that MP7 is a core transcriptional program driving the proliferative state of PRAD malignant cells, which is consistent with the pan‐cancer finding that proliferation is one of the most conserved and prevalent intratumoral heterogeneity programs across solid tumors [7].

Through hdWGCNA, we constructed a single‐cell gene coexpression network and identified the green module that was most significantly correlated with the PRAD‐MP7 signature. By integrating cancer‐specific differential expression analysis and univariate Cox regression, we prioritized YBX1 as the most significant prognostic hazard gene from the overlapping candidate genes. This multistep screening strategy based on single‐cell transcriptional programs ensures that the identified gene is not only a prognostic biomarker but also a functional regulator of the core malignant phenotype in PRAD. We further validated the prognostic value of YBX1 across six independent PRAD cohorts, confirming that high YBX1 expression was consistently associated with shortened OS in the discovery cohort and reduced RFS across multiple validation datasets. These results indicate that YBX1 is a robust and reproducible prognostic biomarker for PRAD, which can be used for risk stratification of PRAD patients in clinical practice.

Our in vitro functional experiments confirmed the pro‐tumorigenic role of YBX1 in PRAD cells. Silencing YBX1 significantly inhibited the viability and proliferation of DU‐145 and PC‐3 cells, as evidenced by CCK‐8 and EdU assays, and downregulated the expression of the proliferation marker Ki‐67. In addition, YBX1 knockdown markedly reduced the mRNA levels of MMP2 and MMP9, two key matrix metalloproteinases that mediate tumor cell invasion and extracellular matrix remodeling [23]. These findings are consistent with previous studies reporting the oncogenic role of YBX1 in other cancer types [24] and, for the first time, demonstrate that YBX1 drives both proliferative and invasive phenotypes in PRAD cells, supporting its role as a functional oncogene in PRAD.

Notably, our study uncovered a pivotal function of YBX1 in modulating the immune landscape of PRAD. Transcriptomic enrichment analyses demonstrated that elevated YBX1 expression was inversely associated with immune activation pathways, particularly those involved in T cell activation and T cell receptor signaling. Moreover, high YBX1 levels correlated with reduced stromal and immune scores, heightened tumor purity, and diminished infiltration of key antitumor immune subsets, including CD8+ cytotoxic T cells, CD4+ T cells, dendritic cells, and neutrophils. These characteristics are consistent with an immune‐excluded “cold” TME, which is widely recognized as a major contributor to the poor response rates observed with immune checkpoint inhibitors in PRAD [25]. In addition, YBX1 expression was positively correlated with that of key immune checkpoint molecules, such as PD‐L1 and CTLA‐4, suggesting that YBX1 may contribute to the formation of a cooperative immunosuppressive microenvironment in PRAD. More importantly, we found that YBX1 could effectively predict immunotherapy response across three independent immunotherapy cohorts, suggesting that it is a potential biomarker for patient stratification in PRAD. It is critical to acknowledge that PRAD is widely recognized as an immunologically “cold” tumor with low objective response rates to single‐agent immune checkpoint blockade (ICB) in standard clinical practice. The three immunotherapy cohorts utilized in this study include patients with advanced solid tumors (including PRAD) treated with ICB, rather than PRAD‐specific cohorts receiving standard‐of‐care ICB monotherapy. Therefore, the predictive performance of YBX1 needs to be further validated in large‐scale, PRAD‐specific, prospective immunotherapy cohorts, particularly in the context of combination immunotherapy strategies currently under clinical investigation for advanced PRAD. Our findings provide a preliminary rationale for YBX1 as a potential stratification biomarker to identify PRAD patients who may benefit from ICB‐based combination therapies, rather than standard single‐agent ICB.

Finally, our drug sensitivity analysis showed that high YBX1 expression was associated with reduced sensitivity to 12 chemotherapeutic and targeted agents, predominantly encompassing cell‐cycle and specific kinase inhibitors (e.g., BMS‐536924, Cediranib, and LGK974). This finding suggests that YBX1 may mediate broad therapeutic resistance in PRAD, which not only explains the poor prognosis of YBX1‐high patients but also provides guidance for personalized treatment selection. For PRAD patients with high YBX1 expression, combination therapies targeting YBX1 may be required to overcome multidrug recalcitrance and improve overall treatment efficacy. Several limitations of this study should be acknowledged. First, our study is mainly based on retrospective public transcriptomic datasets, and the prognostic and predictive value of YBX1 needs to be further validated in large‐scale prospective clinical cohorts. Second, our functional validation is limited to in vitro cell line experiments, and in vivo studies using xenograft models or genetically engineered mouse models are needed to confirm the pro‐tumorigenic and immune‐modulatory roles of YBX1 in PRAD. Third, the precise molecular mechanisms by which YBX1 regulates the proliferative program, immune‐related gene expression, and therapeutic resistance in PRAD remain to be further elucidated, such as its direct downstream target genes and epigenetic regulatory mechanisms.

In conclusion, our integrated multiomics study identifies YBX1 as a key regulator of malignant progression, immune suppression, and therapeutic resistance in PRAD. YBX1 not only serves as a robust prognostic biomarker for risk stratification but also represents a promising therapeutic target for overcoming treatment resistance in PRAD. These findings provide novel insights into the molecular mechanisms of PRAD progression and lay a foundation for the development of YBX1‐targeted therapies for PRAD.

## Author Contributions

K.C. and Z.C. were responsible for project administration and supervision. C.L. was responsible for conceptualization, data curation, formal bioinformatics analysis, in vitro experiments, and writing the original draft. D.L. and J.Y. contributed to methodology, validation, and data visualization.

## Funding

No funding was received for this manuscript.

## Disclosure

All authors read, critically reviewed, and approved the final manuscript for submission.

## Consent

The authors have nothing to report.

## Conflicts of Interest

The authors declare no conflicts of interest.

## Supporting information


**Supporting Information** Additional supporting information can be found online in the Supporting Information section. Supporting Information. Figure S1: Lineage‐specific marker expression heatmap validating the identities of minor tumor microenvironment cell subclusters.

## Data Availability

The transcriptomic and single‐cell RNA sequencing datasets analyzed during the current study are available in public repositories, including TCGA, DKFZ2018, and the Gene Expression Omnibus (GEO) database under the accession numbers GSE107299, GSE21034, GSE70768, GSE70769, and GSE172316. The Tabula Sapiens dataset is available through its dedicated portal. All custom scripts, computational pipelines, and processed analytical data underlying the results reported in this manuscript are available from the corresponding authors upon reasonable request.
