# Supplementary material for: A Single‐Cell Multiomics Pipeline Maps YBX1 as a Functional Biomarker for Immune Evasion and Therapeutic Resistance in Prostate Adenocarcinoma
Source: Hum Mutat. 2026 May 4;2026:2147624. doi: 10.1155/humu/2147624 (PMC13136847; doi:10.1155/humu/2147624)
Supplement: Supplementary file 1 — Supporting Information Additional supporting information can be found online in the Supporting Information section. Supporting Information. Figure S1: Lineage‐specific marker expression heatmap validating the identities of minor tumor microenvironment cell subclusters. [file HUMU-2026-2147624-s001.docx]

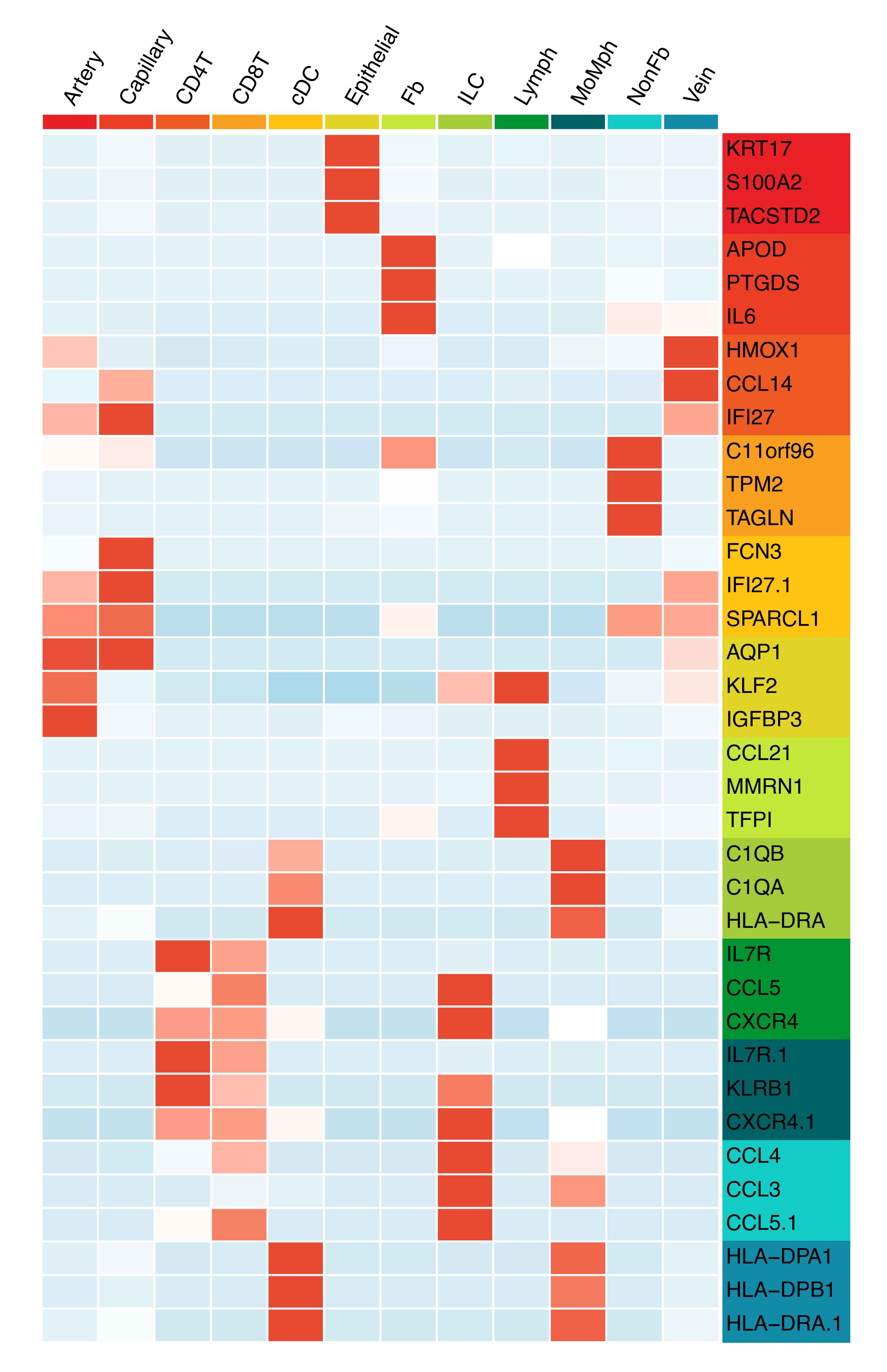


**Figure S1. Lineage-specific marker expression.** Heatmap depicting the expression profiles of canonical marker genes utilized to validate the identities of the minor TME cell subclusters.
